# In vitro antiviral activity of peptide-rich extracts from seven Nigerian plants against three non-polio enterovirus species C serotypes

**DOI:** 10.1186/s12985-021-01628-7

**Published:** 2021-08-04

**Authors:** Omonike O. Ogbole, Toluwanimi E. Akinleye, Abraham O. Nkumah, Aminat O. Awogun, Alfred F. Attah, Moses O. Adewumi, Adekunle J. Adeniji

**Affiliations:** 1 Department of Pharmacognosy, Faculty of Pharmacy, University of Ibadan, Ibadan, Oyo State Nigeria; 2 Department of Pharmacognosy and Drug Development, Faculty of Pharmaceutical Sciences, University of Ilorin, Ilorin, Kwara State Nigeria; 3 Department of Virology, College of Medicine, University of Ibadan, Ibadan, Oyo State Nigeria; 4 WHO Polio National Laboratory, Department of Virology, College of Medicine, University of Ibadan, Ibadan, Oyo State Nigeria

**Keywords:** Antiviral, Circular peptides, Enteroviruses, *Euphorbia hirta*, CPE reduction assay

## Abstract

**Background:**

As frequent viral outbreaks continue to pose threat to public health, the unavailability of antiviral drugs and challenges associated with vaccine development underscore the need for antiviral drugs discovery in emergent moments (endemic or pandemic). Plants in response to microbial and pest attacks are able to produce defence molecules such as antimicrobial peptides as components of their innate immunity, which can be explored for viral therapeutics.

**Methods:**

In this study, partially purified peptide-rich fraction (P-PPf) were obtained from aqueous extracts of seven plants by reverse-phase solid-phase extraction and cysteine-rich peptides detected by a modified TLC method. The peptide-enriched fractions and the aqueous (crude polar) were screened for antiviral effect against three non-polio enterovirus species C members using cytopathic effect reduction assay.

**Results:**

In this study, peptide fraction obtained from *Euphorbia hirta* leaf showed most potent antiviral effect against Coxsackievirus A13, Coxsackievirus A20, and Enterovirus C99 (EV-C99) with IC_50_ < 2.0 µg/mL and selective index ≥ 81. EV-C99 was susceptible to all partially purified peptide fractions except *Allamanda blanchetii* leaf.

**Conclusion:**

These findings establish the antiviral potentials of plants antimicrobial peptides and provides evidence for the anti-infective use of *E. hirta* in ethnomedicine. This study provides basis for further scientific investigation geared towards the isolation, characterization and mechanistic pharmacological study of the detected cysteine-rich peptides.

## Background

The menace caused by viral infections to the health of the public cannot be overstated. Particularly, the frequent outbreaks of newly emerging and re-emerging viruses (from endemic to pandemic situations) coupled with the lack of or limited availability of antiviral drugs and vaccines against them, poses a threat to human survival socio-economically, as evident in the current COVID-19 pandemic [29, 39]. More so, for some viral infections, there is fast development of drug-resistant viral strains due mutation especially, RNA viruses (lacking proof-read mechanisms), and limitation of vaccine use in immunocompromised individuals [28].These have highlighted the need for antiviral drug discovery.

Enteroviruses are non-enveloped icosahedra virion with single-stranded positive sense RNA genome of 7.5 kb size. They belong to 13 species of genus *Enterovirus* in the picornaviridae family, four (EV-A to D) of which have been found to constantly infect humans [9]. Clinical manifestations include aseptic meningitis, neonatal sepsis, myocarditis, type 1 diabetes, hand-foot-and-mouth disease, and acute flaccid paralysis. Poliovirus, the aetiological agent of poliomyelitis is a typical member of enterovirus species C alongside Coxsackievirus A13 (CV-A13), CV-A20, Enterovirus C99 (EV-C99) and others [7, 20].

In Nigeria, circulating vaccine-derived polioviruses (cVDPVs) have been implicated to result from recombination of non-polio enterovirus species C (NPESC) members particularly CV-A13, CV-A20, CV-A11, and CV-A17 with oral polio vaccine (OPV) [1]. The International Health Regulations (IHR) classified Nigeria as a state infected with cVDPVs with potential risk of international spread [12]. Yet, there is currently no available antiviral drugs approved for enterovirus infections.

Peptides, for therapeutic considerations have been faced with concern and limitations such as poor pharmacokinetic properties, and high molecular weight (immunogenicity) [17, 23, 24, 31, 45]. Some techniques such as cyclization, incorporation of unnatural amino acids, recombinant techniques have been employed to enhance properties of target peptides [17]. Diverse peptides are produced by plants for various metabolic purposes including defence against attacks from microbes, herbivores and pests [8]. As plants continue to be a veritable source for drug discovery, the presence of cysteine-rich peptides including the circular variants in plants and particularly, cysteine-rich circular peptides known as cyclotides, brightens the future of peptide drug discovery. Of the five structural groups of antimicrobial plant peptides [18], cyclotides are found to be ultra-stable, being able to withstand extreme conditions of temperature, chemical, and enzymatic treatment [2, 16].

Viral therapeutic peptides are emerging [11], yet plant-derived peptides have not been explored for antiviral activity. Herein, we evaluated the antiviral effect of partially-purified peptide fraction (P-PPf) from seven medicinal plants belonging to Rubiaceae, Euphorbiaceae, Phyllantaceae, and Apocynaceae families against 3 members of NPESC.

## Methods

### Plants material collection, authentication and peptide extraction

Leaf part of 3 plants from Euphorbiaceae, 1 from Rubiaceae, 1 from Phyllantaceae and 2 from Apocynaceae were collected from the Botanical Garden of [BLINDED FOR PEER REVIEW], identified and authenticated at Forestry Herbarium Ibadan (FHI). Leaves were air-dried, pulverized and subjected to aqueous and then solid-phase extractions. Extraction method was employed in view of cyclotides, using previously described procedures [8, 14–16]. Briefly, plants leaves were subjected to aqueous extraction by maceration in dichloromethane/methanol (1:1; v/v) for 24 h at 25 °C with continuous agitation. After 24 h, water was added to obtain aqueous-rich fraction. The concentrated aqueous-rich fraction was further subjected to reverse-phase solid-phase extraction (RP-SPE) using C_18_ columns (Phenomenex, Aschaffenburg, Germany) and eluted with solvent B (90% (v/v) acetonitrile, 0.045% (v/v) trifluoroacetic acid in double distilled water). Hydrophilic compounds were separated from partially purified peptide fraction (P-PPf) by eluting with 20% and 80% solvent B, respectively. The P-PPfs were freeze-dried and stored in the refrigerator at 4 °C until used for bioassay.

### Thin layer chromatography (TLC) chemical detection of peptides

A modified method previously described by WenYan et al*.* [48] and Attah et al*.* [2] was adopted for the TLC chemical detection. Pre-coated TLC plates (G_254_ MERCK, Germany) and solvent system *n*-butanol:acetic acid:water (3:1:1) were used. Each solvent-dissolved peptide extract was spotted on the TLC plate and developed in the solvent system above. Plates were allowed to dry, viewed under UV at 254 and 365 nm. Dried plates (TLC chromatograms) were swiftly sprayed or dipped in freshly prepared G-250 modified stain or ninhydrin, respectively.

### Preparation of extract stock

For antiviral screening, 20 mg of fractions (crude and peptide-rich) was each dissolved in 2 mL dimethylsulfxoide (DMSO) to obtain stock solutions (10 mg/mL).

### Cell and virus

Human breast adenocarcinoma cancer cell line (MCF-7) obtained from WHO national Polio Lab, Ibadan, Nigeria was used for both cytotoxic and antiviral studies. Cells were grown in Eagle’s minimum essential medium (MEM) supplemented with 10% foetal bovine serum (FBS), 100 units/mL of penicillin, 100 μg/mL of streptomycin, 2 mM L-glutamine, 0.07% NaHCO3, 1% non-essential amino acids and vitamin solution at 37 °C in a humidify incubator (85–95% humidity). Three species C enterovirus members, including two serotypes of coxsackie virus A (CV-A13 and CV-A20) and a numbered Enterovirus C serotype (EV-C99) were obtained from stool isolates [9] by the Enterovirus research group, Department of Virology, [BLINDED FOR PEER REVIEW]. The test medium used for cytotoxic assays and antiviral assays contained only 2% FBS.

### Preparation of viral stocks

To increase the quantity of virion stocks, virus suspension (200 µL) was inoculated into the T25 flask of cultured MCF-7 cells, and incubated at 37 °C for about 72 h for 100% cytopathic effect. Afterwards, medium was centrifuged and aliquots of supernatant were made into cryovials. All viral stocks were stored at − 70 °C until use.

### Tissue culture infective dose (TCID_50_)

Virus titre was determined by virus-induced cytopathic effect (vCPE) in MCF-7 cell and were expressed as 50% tissue culture infective concentration (TCID_50_) per mL. Briefly, 100 µL MCF-7 cell suspension (1 × 10^5^ cells/mL) was seeded into a 96-well microtitre plate and incubated for 24 h to form monolayer. Afterward, virus suspension (100 µL) was inoculated into the eight wells (as replicates) of each column 1–10 with varying (ten-fold serially diluted- 10^–1^ to 10^–10^) concentration per column. Column 11 and 12 served as the cell control. Plate was incubated at 37 °C, and daily CPE scoring was done for about 7 days when cell control wells started dying off. The TCID_50_ values were determined using Spearman–Karber’s method and 100 TCID_50_ was used for the antiviral assay.

### Cytotoxicity assay

The maximum nontoxic concentration (MNTC) test of crude fractions to MCF-7 cells in culture was determined by 3-(4,5-dimethylthiazol-2-yl)-2,5-diphenyltetrazolium bromide (MTT, Sigma Aldrich®) assay, a colorimetric assay that reliably measures cell viability. Previously described method by Mossmann [32] was adopted. Briefly, previously seeded monolayers of MCF-7 cells in a 96-well microtitre plate was treated with six serial ten-fold dilutions (1000 to 0.01 μg/mL) of stock solutions of crude and peptide-rich fractions in maintenance medium (2% MEM) for 72 h. Afterwards, plates were observed for MNTC on the cells under an inverted microscope (OLYMPUS CKX31). Afterward, old medium was removed and 25 μL of prepared MTT reagent in phosphate buffer saline (PBS) (2 mg/mL) was added to each well, including controls and plate returned to the incubator for 2 h. Then, DMSO (75 μL) was added to solubilize the formazan crystals formed. Optical density values were obtained by spectrophotometry (Multiscan 347, MTX lab) at 490 nm. Data obtained was used to determine 50% cytotoxic concentration (CC_50_).

### Virus-induced cytopathic effect (vCPE) reduction assay

Previously described neutralization method [27, 40] was employed to evaluate the antiviral vCPE inhibition effects of pre-purified peptide fractions on the three species C enteroviruses. Concisely, six serial two-fold dilutions made from the MNTC of each of the fractions was added to confluent cell monolayers in a 96-well plate, and allowed to adsorb for about 1 h at 37 °C, after which 100 TCID_50_ virus suspension was added. Plates were incubated at 37 °C for 72 h (plant fractions were kept during incubation). Positive control (virus control) wells were infected with the same concentration of virus but untreated with fractions, while negative (cell control) wells contained only maintenance medium (uninfected and untreated cell). Plates was observed preliminarily under the microscope for vCPE. Thereafter, MTT colorimetric measure was employed as described earlier. The concentration that reduced 50% of CPE with respect to the virus control was defined as the 50% inhibitory concentration (IC_50_). Since there are no approved antiviral drugs for enterovirus infections, no standard drug was used.

### Data analysis

#### Selective index, CC_50_ and IC_50_

The 50% cytotoxic concentration (CC_50_) and the 50% inhibitory concentration (IC_50_) for each extract was calculated from non-linear regression analysis using GraphPad prism5. The selective index, which is the index of safety margin is defined as CC_50_ over IC_50_.

## Results

### Thin layer chromatography (TLC) chemical detection of cysteine-rich peptides

**Fig. 1:**
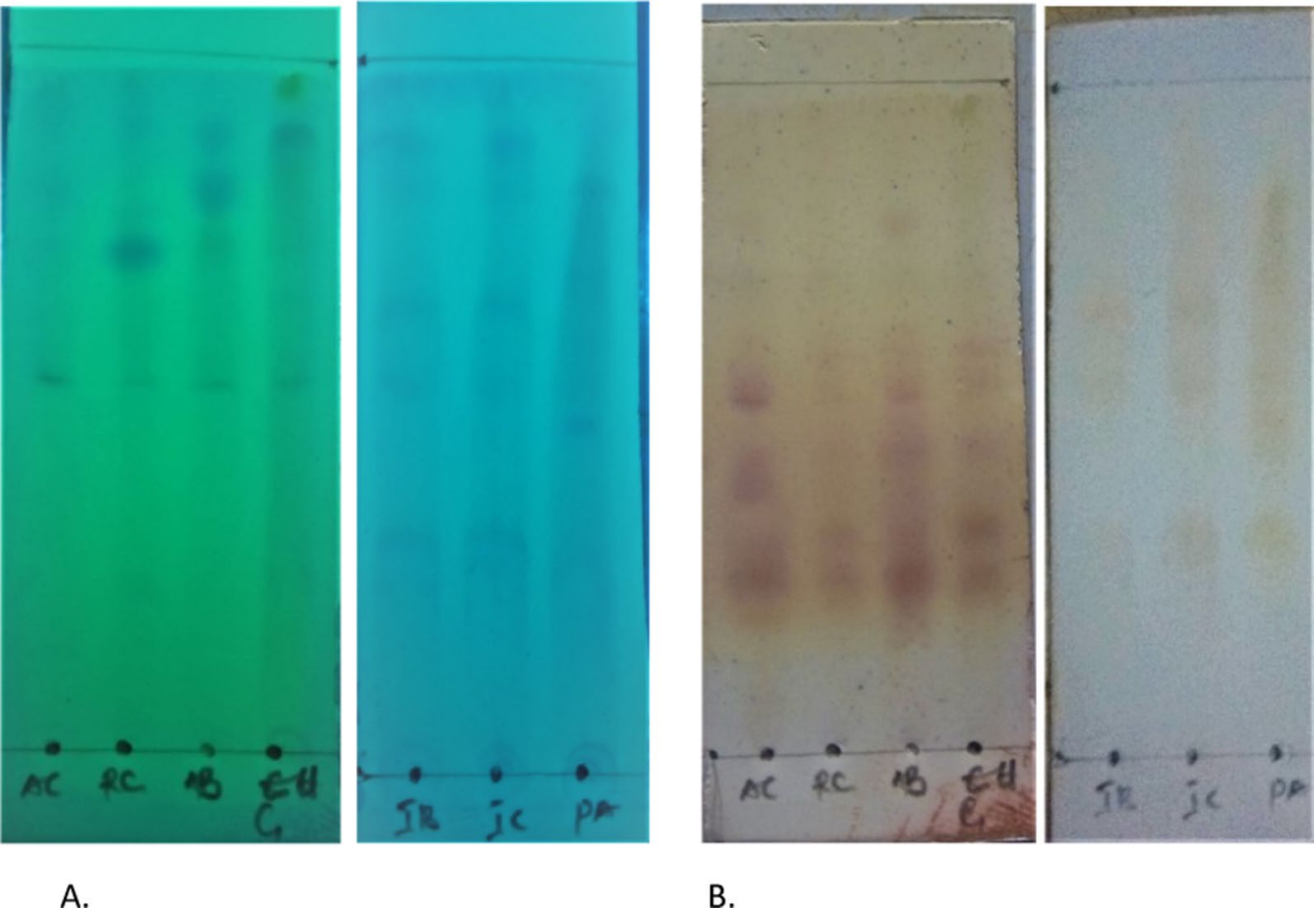
Chromatogram showing the chemical detection of peptides from plants using **a** modified G250 stain and **b** Ninhydrin. *Allamanda blanchetii* = AB; *Allamanda cathartica* = AC; *Euphorbia Gramineae* = RC; *Euphorbia hirta* = IR; *Euphorbia humifusa* = EH; *Phyllanthus amarus* = PA; Ixora coccinea = IC

The bound P-PPf was eluted from the aqueous-rich fraction by reverse-phase solid-phase extraction (RP-SPE) using C_18_ columns (Phenomenex, Aschaffenburg, Germany). On spraying with freshly prepared G-250 modified stain, all partially purified peptide fraction spotted on TLC pre-coated plates produced a bright blue colouration indicating the presence of cysteine-rich peptides which may be circular in their configuration (Fig. 1a). Furthermore, on spraying with ninhydrin (which characterizes presence of amino acids, amines and linear peptide by colour change from purple to red) Ninhydrin presented colour changes indicative of the presence of peptides, likely a combination of linear and circular peptides if present (Fig. 1b).

### Tissue culture infective dose (TCID_50_)

As determined by Spearman–Karber’s method, the virus titre for CV-A13 and EV-C99 gave the value of 10^–4^ with 100 TCID_50_ calculated as 10^–2^, while CV-A20 a virus titre value of 10^–3^ with 100 TCID_50_ calculated to be 10^–1^.

### Cytotoxic activities of crude and pre-purified peptide fractions

The aqueous crude and P-PPf of each plant both had equal MNTC value in MCF-7 cells. All the tested fractions had a common MNTC value of 10 µg/mL, except for *Allamanda* *blanchetii* and *Euphorbia* *humifusa* (100 µg/mL) (Table 1). As shown by the CC_50_ values (Table 2), the peptide fraction of *Ixora coccinea* (ICp) relatively had the highest cytotoxicity (19.7 μg/mL) followed by the peptide of *Allamanda* *cathartica* (20.5 μg/mL), while the peptide fraction of *Euphorbia humifusa* (EHu) had the lowest (169.0 μg/mL).

**Table 1:** Plants species evaluated for antiviral activity

S/N	Family	Name	Code	Voucher (FHI) No	MNTC (µg/mL)
1	Apocynaceae	*Allamanda blanchetii* A.DC	AB	112880	100
2	Apocynaceae	*Allamanda cathartica* L	AC	112881	10
3	Euphorbiaceae	*Euphorbia graminea* Jacq	EG	112894	10
4	Euphorbiaceae	*Euphorbia hirta* L	EHi	112893	10
5	Euphorbiaceae	*Euphorbia humifusa* Willd	EHu	112948	100
6	Phyllantaceae	*Phyllanthus amarus* Schumach. & Thonn	PA	112892	10
7	Rubiaceae	*Ixora coccinea* L	IC	112882	10

**Table 2:** Antiviral Activity of crude aqueous fraction and partially purified peptide fractions on three NPESC members

Extract	CC_50_ (µg/mL)	CV-A13		CV-A20		EV-C99	
		IC_50_ (µg/mL)	SI	IC_50_ (µg/mL)	SI	IC_50_ (µg/mL)	SI
ABp	167.2	33.31	5.0	NA	NA	NA	NA
ACc	24.3	NA	NA	2.67	9.1	1.64	14.8
ACp	20.5	NA	NA	1.09	18.8	1.18	17.3
ICc	109.8	NA	NA	1.75	62.9	1.85	59.5
ICp	19.7	NA	NA	0.54	36.4	0.54	36.8
EHic	81.6	2.81	29.0	1.65	49.3	0.80	102.5
EHip	159.0	0.94	169.0	1.96	81.0	0.53	301.7
EHuc	167.5	17.28	9.7	19.57	8.6	9.45	17.7
EHup	169.0	20.94	8.1	5.47	30.9	5.30	31.9
EGc	33.2	1.969	16.8	NA	NA	0.644	51.6
EGp	39.1	3.203	12.2	1.663	23.5	0.531	73.6
PAp	68.0	NA	NA	1.11	61.5	0.59	116.3

c—crude aqueous fraction; p—partially purified peptide fraction; NA—not active; SI—selective index; CC50—50% cytotoxic concentration; IC50—50% inhibitory concentration; CVA13—Coxsackievirus A13; CVA20—Coxsackievirus A20; EVC99—Enterovirus C99

### Antiviral screening of crude and peptide fractions

All tested fractions showed considerable antiviral activity variably on the three viruses (Table 2). Also, all P-PPfs showed antiviral activity across the three NPESC members except for *Allamanda* *blanchetii*, *Allamanda* *cathartica*, *Phyllanthus* *amarus*, and *Ixora* *coccinea*. In general, the antiviral activity of crude and peptide fractions of each plant is consistent, with enhanced effect observed with the peptide fractions.

## Discussion

Historically, medicinal plants have been a valuable source for drug discovery. Plant peptides are gaining attention for drug discovery exploration especially, cysteine-rich circular peptides due to their stability [3, 11, 50]. Antimicrobial function of plant peptides in plant innate immunity can be explored for antiviral drug discovery [3, 16]. Though poliovirus infection is on the edge of eradication, there is need to search for antivirals against nonpolio enteroviruses that can substitute the niche as the leading cause of paralysis in children [5].

In this study, all tested pre-purified peptide fractions from the *Euphorbia* species notably showed antiviral effect across all the NPESC serotypes. *Euphorbia hirta* evidently showed best activity with IC_50_ (≤ 2 µg/mL) and high index of safety margins (SI ≥ 81). Members of Euphorbiaceae family especially, *Euphorbia* species extract have been demonstrated for in vitro antiviral activity against RNA and DNA viruses [10, 13, 21, 22, 25, 37, 38, 40, 42, 44, 51]. Also, various in vitro antiviral activities against hepatitis B, herpes simplex virus, influenza viruses, rhinovirus, and enterovirus [4, 6, 30, 33, 43, 46] have been displayed by some small molecules from *Euphorbia*. Thus, this finding is consistent with reports on antiviral potentials of *Euphorbia* species. Among the three *Euphorbia* species tested, *E. hirta* was observed to show best antiviral activity across the three NPESC serotypes with its p-PPf exerting highly selective antiviral activity, more enhanced than its crude fraction; which is further evident in the relatively higher selective index values of P-PPf of *E. hirta* (Table 2). *E. hirta* has been documented in ethnomedicine use against infections including viral infections in Philippines, India, Pakistan and Sri Lanka [41]. Similar peptides with varying proportion or varying peptide constituents in the tested *Euphorbia* species could be responsible for their unequal antiviral activity. Ongoing process of isolation and characterization of the peptides will reveal this clearly.

Partially purified peptide fractions from *Allamanda* *blanchetii* showed moderate antiviral effect only on CV-A13 while *Allamanda* *cathartica* lacked antiviral effect only on CV-A13. This varying antiviral effects of the two *Allamanda* species observed across the three NPESC serotypes could suggest disparate peptide constituents in the two species. Nguyen and his group reported the presence of allotides, proline-rich cystine knot α-amylase inhibitors from *Allamanda cathartica*; the extremely stable disulphide-rich peptides with alpha amylase activity and poor antimicrobial activity [36].

The antiviral assay design was prophylactic and not therapeutic. Thus, possible mechanism of antiviral action could be the prevention of virus attachment/entry into susceptible MCF-7 cell line used or inhibition of a replication stage that is downstream of entry or direct effect on virion (virucidal). CV-A13 and CV-A20 use cell surface receptor intercellular adhesion molecule 1 (ICAM-1) for entry into susceptible cells [19], thus binding of peptides to the glycoprotein ICAM-1 is a possible antiviral target. However, alternate cell entry have been documented for CV-A20 other than ICAM-1 [34], indicating the differing results for some partially purified peptides exhibiting antiviral activity on CV-A13 and not on CV-A20. Plant-derived cysteine knot peptides include alpha amylase inhibitors, cyclotides, thionins, and defensins whose bioactivities lead to blocking of viral infection by clustering the viral particles and blocking receptor binding [35, 47]. These disulphide stabilised peptides mediate in the inhibition of viral entry, viral particle disruption, interference with essential cell signalling or viral gene expression [26], or by other poorly-understood mechanisms. In addition to the antiviral activities, cysteine-rich peptides such as defensins modulate adaptive immune responses via mobilization of dendritic cells, induction of their maturation, enhancement of antigen uptake, and mobilization of T Lymphocytes (CD4 + and CD8 + effector T cells) to sites of infection, due to the T cell-chemoattracting effect of defensins [47, 49].

## Conclusion

Semi-purified cysteine-rich peptides in the tested *Euphorbia* species displayed notable antiviral activity against non-polio enterovirus species C; CV-A13, CV-A20 and EV-C99 in MCF-7 cell culture system. To the best of our knowledge, this is the first antiviral report on semi-purified peptides from the tested plant species and therefore provides scientific rationale for a more extensive study of the individual peptides, molecular targets, safety and efficacy as potential peptide-based therapeutics.

## Data Availability

Available from the corresponding author, upon reasonable request.

## Abbreviations

FHI: Forestry Herbarium Ibadan
cVDPVs: Circulating vaccine-derived polioviruses
NPESC: Non-polio enterovirus species C
OPV: Oral polio vaccine
IHR: The International Health Regulations
P-PPf: Partiallypurified peptide fraction
TLC: Thin layer chromatography
UV: Ultraviolet
DMSO: Dimethylsulfoxide
WHO: World Health Organization
MEM: Minimum essential medium
FBS: Fetal bovine serum
vCPE: Virus-induced cytopathic effect
TCID: Tissue culture infective dose
MNTC: Maximum non-toxic concentration
BSAA: Broad-spectrum antiviral activity
ICAM: Intercellular adhesion molecule

## References

[1] Adeniji AJ, Faleye TCO (2014). Impact of cell lines included in enterovirus isolation protocol on perception of nonpolio enterovirus species c diversity. J Virol Methods.

[2] Attah AF, Hellinger R, Sonibare MA, Moody JO, Arrowsmith S, Wray S, Gruber CW (2016). Ethobotanical survey of *Rinorea dentata* (violaceae) used in South-Western Nigerian ethnomedicine and detection of cyclotides. J Ethnopharmacol.

[3] Broekaert WF, Cammue BPA, De Bolle MFC, Thevissen K, De Samblanx GW, Osborn RW (1997). Antimicrobial peptides from plants. Crit Rev Plant Sci.

[4] Chang SY, Park JH, Kim YH, Kang JS, Min J-Y (2016). A natural component from *Euphorbia humifusa* willd displays novel, broad-spectrum anti-influenza activity by blocking nuclear export of viral ribonucleoprotein. Biochem Biophys Res Commun.

[5] Chard AN, Datta SD, Tallis G, Burns CC, Wassilak SG, Vertefeuille JF, Zaffran M (2020). Progress toward polio eradication—worldwide, January 2018–March 2020. Morb Mortal Wkly Rep.

[6] Cheng H-Y, Lin T-C, Yang C-M, Wang K-C, Lin L-T, Lin C-C (2004). Putranjivain a from *Euphorbia jolkini* inhibits both virus entry and late stage replication of herpes simplex virus type 2 in vitro. J Antimicrob Chemother.

[7] Dufresne AT, Gromeier M (2004). A nonpolio enterovirus with respiratory tropism causes poliomyelitis in intercellular adhesion molecule 1 transgenic mice. Proc Natl Acad Sci.

[8] Fahradpour M, Keov P, Tognola C, Perez-Santamarina E, Mccormick PJ, Ghassempour A, Gruber CW (2017). Cyclotides isolated from an ipecac root extract antagonize the corticotropin releasing factor type 1 receptor. Front Pharmacol.

[9] Faleye T, Adewumi M, Japhet M, David O, Oluyege A, Adeniji J, Famurewa O (2017). Non-polio enteroviruses in faeces of children diagnosed with acute flaccid paralysis in Nigeria. Virol J.

[10] Faral-Tello P, Mirazo S, Dutra C, Pérez A, Geis-Asteggiante L, Frabasile S, Koncke E, Davyt D, Cavallaro L, Heinzen H (2012). Cytotoxic, virucidal, and antiviral activity of South American plant and algae extracts. Sci World J.

[11] Findlay EG, Currie SM, Davidson DJ (2013). Cationic host defence peptides: potential as antiviral therapeutics. BioDrugs.

[12] GPEI-WHO. Polio this week in nigeria. March 2019 ed. Global Polio Eradication Initiative, World Health Organization, Geneva. 2019. www.polioeradication.org/where-we-work/nigeria/. Accessed 20 April 2020.

[13] Gyuris A, Szlavik L, Minarovits J, Vasas A, Molnar J, Hohmann J (2009). Antiviral activities of extracts of *Euphorbia hirta* l. Against HIV-1, HIV-2 and SIVMAC251. In Vivo.

[14] Hashempour H, Koehbach J, Daly NL, Ghassempour A, Gruber CW (2013). Characterizing circular peptides in mixtures: Sequence fragment assembly of cyclotides from a violet plant by maldi-tof/tof mass spectrometry. Amino Acids.

[15] Hellinger R, Koehbach J, Puigpinos A, Clark RJ, Tarrago T, Giralt E (2015). Inhibition of human prolyl oligopeptidase activity by the cyclotide psysol 2 isolated from *Psychotria solitudinum*. J Nat Prod.

[16] Hellinger R, Koehbach J, Soltis DE, Carpenter EJ, Wong GK-S, Gruber CW (2015). Peptidomics of circular cysteine-rich plant peptides—analysis of the diversity of cyclotides from viola tricolor by transcriptome- and proteome-mining. J Proteome Res.

[17] Henninot A, Collins JC, Nuss JM (2018). The current state of peptide drug discovery: back to the future?. J Med Chem.

[18] Hiemstra PS, Zaat SJ (2013). Antimicrobial peptides and innate immunity.

[19] Jonsson N, Gullberg M, Israelsson S, Lindberg AM (2009). A rapid and efficient method for studies of virus interaction at the host cell surface using enteroviruses and real-time PCR. Vir J.

[20] Kapoor A, Ayyagari A, Dhole T (2001). Non-polio enteroviruses in acute flaccid paralysis. Ind J Pediatr.

[21] Karimi A, Mohammadi-Kamalabadi M, Rafieian-Kopaei M, Amjad L (2016). Determination of antioxidant activity, phenolic contents and antiviral potential of methanol extract of *Euphorbia spinidens* bornm (euphorbiaceae). Trop J Pharm Res.

[22] Lam WY, Leung KT, Law PTW, Lee SMY, Chan HLY, Fung KP, Ooi VEC, Waye MMY (2006). Antiviral effect of *Phyllanthus nanus* ethanolic extract against Hepatitis B virus (HBV) by expression microarray analysis. J Cell Biochem.

[23] Lau JL, Dunn MK (2017). Therapeutic peptides: historical perspectives, current development trends, and future directions. Bioorg Med Chem.

[24] Lee AC-L, Harris JL, Khanna KK, Hong J-H (2019). A comprehensive review on current advances in peptide drug development and design. Int J Mol Sci.

[25] Lin C-C, Cheng H-Y, Yang C-M, Lin T-C (2002). Antioxidant and antiviral activities of *Euphorbia thymifolia* l. J Biomed Sci.

[26] Lin S, Liu M, Wang S, Li S, Yang Y, Shi J (2008). Coumarins from branch of *Fraxinus sieboldiana* and their antioxidative activity. Zhongguo Zhong yao za zhi = Zhongguo zhongyao zazhi = China J Chin Mater Med.

[27] Lin Y-J, Chang Y-C, Hsiao N-W, Hsieh J-L, Wang C-Y, Kung S-H, Tsai F-J, Lan Y-C, Lin C-W (2012). Fisetin and rutin as 3c protease inhibitors of enterovirus A71. J Virol Methods.

[28] Ljungman P (2012). Vaccination of immunocompromised patients. Clin Microbiol Infect.

[29] Maria N, Zaid A, Catrin S, Ahmed K, Ahmed A, Christos I, Maliha A, Riaz A (2020). The socio-economic implications of the coronavirus pandemic (COVID-19): a review. Int J Surg.

[30] Madureira A, Ascenso J, Valdeira L, Duarte A, Frade J, Freitas G, Ferreira M (2003). Evaluation of the antiviral and antimicrobial activities of triterpenes isolated from *Euphorbia segetalis*. Nat Prod Res.

[31] Morrison C (2018). Constrained peptides’ time to shine?. Nat Rev Drug Disc.

[32] Mosmann T. Rapid colorimetric assay for cellular growth and survival: application to proliferation and cytotoxicity assays. J Immunol Methods. 1983;65(1-2):55–63. 10.1016/0022-1759(83)90303-4 6606682

[33] Mucsi I, Molnár J, Hohmann J, Rédei D (2001). Cytotoxicities and anti-herpes simplex virus activities of diterpenes isolated from euphorbia species. Planta Med.

[34] Newcombe NG, Andersson P, Johansson ES, Au GG, Lindberg AM, Barry RD, Shafren DR (2003). Cellular receptor interactions of c-cluster human group a coxsackieviruses. J Gen Virol.

[35] Nguyen KNT, Nguyen GKT, Nguyen PQT, Ang KH, Dedon PC, Tam JP (2016). Immunostimulating and gram-negative-specific antibacterial cyclotides from the butterfly pea (*Clitoria ternatea*). FEBS J.

[36] Nguyen PQ, Luu TT, Bai Y, Nguyen GK, Pervushin K, Tam JP (2015). Allotides: proline-rich cystine knot α-amylase inhibitors from *Allamanda cathartica*. J Nat Prod.

[37] Nothias-Scaglia L-F, Dumontet V, Neyts J, Roussi F, Costa J, Leyssen P, Litaudon M, Paolini J (2015). Lc-ms2-based dereplication of euphorbia extracts with anti-chikungunya virus activity. Fitoterapia.

[38] Notka F, Meier G, Wagner R (2003). Inhibition of wild-type human immunodeficiency virus and reverse transcriptase inhibitor-resistant variants by *Phyllanthus amarus*. Antivir Res.

[39] Obi S, Yunusa T, Ezeogueri-oyewole A, Sekpe S, Egwemi E, Isiaka A (2020). The socio-economic impact of covid-19 on the economic activities of selected states in Nigeria. Indones J Soc Environ Issue.

[40] Ogbole OO, Akinleye TE, Segun PA, Faleye TC, Adeniji AJ (2018). In vitro antiviral activity of twenty-seven medicinal plant extracts from southwest Nigeria against three serotypes of echoviruses. Virology J.

[41] Perera SD, Jayawardena UA, Jayasinghe CD (2018). Potential use of *Euphorbia hirta* for dengue: a systematic review of scientific evidence. J Trop Med.

[42] Ramezani M, Behravan J, Arab M, Farzad SA (2008). Antiviral activity of *Euphorbia helioscopia* extract. J Biol Sci.

[43] Tian Y, Sun L-M, Li B, Liu X-Q, Dong J-X (2011). New anti-HBV caryophyllane-type sesquiterpenoids from *Euphorbia humifusa* willd. Fitoterapia.

[44] Torky ZA (2016). Antiviral activity of *Euphorbia* lectin against herpes simplex virus 1 and its antiproliferative activity against human cancer cell-line. J Antivir Antiretrovir.

[45] Vilas Boas LCP, Campos ML, Berlanda RLA, De Carvalho NN, Franco OL (2019). Antiviral peptides as promising therapeutic drugs. Cell Mol Life Sci.

[46] Wang B, Wei Y, Zhao X, Tian X, Ning J, Zhang B, Deng S, Li D, Ma X, Wang C (2018). Unusual ent-atisane type diterpenoids with 2-oxopropyl skeleton from the roots of *Euphorbia ebracteolata* and their antiviral activity against human rhinovirus 3 and enterovirus 71. Bioorg Chem.

[47] Weber F, Bamford DH, Zuckerman M (2021). Antiviral innate immunity: introduction. Encyclopedia of virology.

[48] Wenyan X, Jun T, Changjiu J, Wenjun H, Ninghua T (2008). Application of a TLC chemical method to detection of cyclotides in plants. Chin Sci Bull.

[49] Yang D, Liu ZH, Tewary P, Chen Q, de la Rosa G, Oppenheim JJ (2007). Defensin participation in innate and adaptive immunity. Curr Pharm Des.

[50] Zasloff M (2002). Antimicrobial peptides of multicellular organisms. Nature.

[51] Zheng W, Cui Z, Zhu Q (1998). Cytotoxicity and antiviral activity of the compounds from euphorbia kansui. Planta Med.

